# Acute Pancreatitis Associated With Semaglutide in a Patient With Multimorbidity: A Case Report

**DOI:** 10.7759/cureus.101908

**Published:** 2026-01-20

**Authors:** Shao Chi Lo, Hsing Jung Yeh

**Affiliations:** 1 Department of Internal Medicine, Division of Gastroenterology and Hepatology, Taipei Medical University Hospital, Taipei, TWN

**Keywords:** acute pancreatitis (ap), glp-1 ra, multimorbidity pattern, semaglutide, type 2 diabetes

## Abstract

Semaglutide, a long-acting glucagon-like peptide-1 receptor agonist (GLP-1 RA), has shown robust efficacy in glycemic control, weight reduction, and cardiovascular protection, but concerns remain regarding rare adverse events such as acute pancreatitis. We report a 59-year-old man with type 2 diabetes, stage IIIA colon cancer post-hemicolectomy and chemotherapy, stage 4 chronic kidney disease, hypertension, and dyslipidemia who developed acute pancreatitis after 2.5 years of weekly semaglutide therapy. He presented with epigastric pain and elevated lipase, while abdominal ultrasound revealed only mild pancreatic parenchymal heterogeneity without peripancreatic fluid collection. The patient improved with conservative management, and semaglutide was discontinued in favor of linagliptin, with no recurrence of symptoms during three months of follow-up. This case highlights the potential risk of pancreatitis in patients with multimorbidity, a population frequently excluded from randomized controlled trials and underrepresented in real-world prescribing due to socioeconomic barriers. Clinicians should remain vigilant when prescribing semaglutide to patients with multiple underlying chronic conditions, and further studies focusing on multimorbid populations are warranted to clarify its safety profile.

## Introduction

Semaglutide is a long-acting glucagon-like peptide-1 receptor agonist (GLP-1 RA) with 94% sequence homology to native human GLP-1. It improves glycemic control through glucose-dependent insulin secretion, suppression of inappropriate glucagon release, delayed gastric emptying, and appetite reduction [1]. Owing to these effects, semaglutide has become widely prescribed for type 2 diabetes mellitus, weight reduction, and cardiovascular risk reduction [2-4].

Despite its established efficacy, concerns remain regarding its gastrointestinal adverse effects [5]. While nausea, vomiting, diarrhea, constipation, and abdominal pain are the most frequently reported side effects, rare but clinically significant complications such as acute pancreatitis have also been described [6,7]. Large randomized controlled trials and meta-analyses have generally not demonstrated an increased overall risk of acute pancreatitis with semaglutide compared with placebo; however, these trials often enrolled highly selected patient populations [8].

Importantly, patients with advanced chronic kidney disease, recent malignancy, or multiple comorbidities are frequently excluded from pivotal clinical trials, leaving uncertainty regarding drug safety in real-world, high-risk populations. It remains unclear whether certain subgroups, such as patients with multimorbidity - commonly defined as the coexistence of two or more chronic conditions within the same individual - may be at higher risk. Several mechanisms have been proposed to explain a potential association between GLP-1 receptor agonists and pancreatitis, including modulation of exocrine pancreatic secretion, altered pancreatic ductal dynamics secondary to delayed gastric emptying, and increased pancreatic enzyme concentration due to reduced clearance [9]. Nevertheless, causality remains incompletely understood.

Here, we report a case of acute pancreatitis occurring after long-term semaglutide therapy in a patient with multiple comorbidities, highlighting the importance of cautious risk assessment and post-marketing vigilance when prescribing GLP-1 receptor agonists in complex clinical settings.

## Case presentation

A 59-year-old man had a past medical history significant for stage IIIA ascending colon cancer, type 2 diabetes mellitus, hypertension, stage 4 chronic kidney disease, and dyslipidemia. The patient had a smoking history of over 40 years, averaging approximately 20 cigarettes per day, and he had a history of alcohol consumption but had remained abstinent for the preceding five years. Past surgical history was significant for a laparoscopic right hemicolectomy three years ago for colon cancer. He finished the adjuvant chemotherapy with 10 courses of the FOLFOX6 regimen (including leucovorin calcium, fluorouracil, and oxaliplatin) between November 3, 2022, and March 17, 2023. The patient had no prior history of acute or chronic pancreatitis.

At our outpatient clinic, the patient was on long-term medications, and his regimen included aspirin, bisoprolol, valsartan, amlodipine, and rosuvastatin for cardiovascular disease; glimepiride and weekly subcutaneous semaglutide 1.0 mg for type 2 diabetes; and multiple agents for chronic kidney disease, including sodium bicarbonate, calcium polystyrene sulfonate, ketoanalogues of essential amino acids, folic acid, and intermittent darbepoetin alfa.

The patient had a history of type 2 diabetes mellitus for more than 25 years. Three years prior to presentation, dulaglutide was initiated in combination with metformin for glycemic control. However, after six months of dulaglutide therapy, his HbA1c level remained elevated at 7.6%. At that time, his body weight was 57.5 kg, and his height was 158 cm, with a body mass index (BMI) of 23.03 kg/m^2^. Consequently, semaglutide was prescribed by his outpatient physician to achieve better glycemic management. By the time of his emergency department visit, he had been receiving semaglutide therapy for 2.5 years, during which his HbA1c levels were maintained within the range of 5.6% to 6.6%. Semaglutide was initiated at 0.25 mg subcutaneously once weekly and was gradually titrated to 1.0 mg once weekly, which he continued on a long-term basis. There had been no recent dose escalation, interruption, or reinitiation of semaglutide prior to the onset of acute pancreatitis.

He presented to the emergency department in our hospital at night with the complaint of epigastric pain that had begun in the afternoon and had persisted for approximately six hours. The pain was intermittent, with an intensity of 4-5 on a numerical rating scale, and was not relieved by positional changes or radiating to the shoulder or back. The patient also described cold sweating. He denied fever, chest pain, as well as nausea, vomiting, diarrhea, alcohol consumption, tobacco use, or recreational drug use.

All vital signs were in normal range, and the physical examination revealed normal breath sounds, a regular heart rhythm, and a flat, non-tender abdomen. Laboratory investigations showed normocytic anemia, with a hemoglobin level of 10.0 g/dL, and the platelet and leukocyte counts were within normal limits. Biochemical testing demonstrated significant renal impairment, with a serum creatinine of 5.3 mg/dL and an estimated glomerular filtration rate (eGFR) of 12 mL/min/1.73 m^2^. Hepatic enzymes were elevated, including aspartate aminotransferase (AST) at 68 U/L and alanine aminotransferase (ALT) at 71 U/L. The serum total bilirubin was 0.2 mg/dL. Lactate dehydrogenase (LDH) was also elevated at 264 IU/L. The serum lipase was markedly increased at 367 U/L. Electrolytes showed hypocalcemia, with sodium 136 mEq/L, potassium 5.1 mEq/L, and adjusted calcium 7.0 mg/dL. Random blood glucose was 114 mg/dL. Hemoglobin A1C was measured to be 5.8%, and triglycerides were normal at 132 mg/dL. The immunoglobulin G4 (IgG4) subclass was 15.4 mg/dL (Table 1). The computed tomography for abdomen with contrast was not arranged due to his acute-on-chronic kidney disease. Based on the clinical symptoms of abdominal pain and elevated lipase levels, he was diagnosed with acute pancreatitis and admitted to our general ward for treatment.

**Table 1:** Laboratory results at initial presentation WBC: white blood cell count; MCV: mean corpuscular volume; HGB: hemoglobin; PLT: platelet count; PT: prothrombin time; INR: international normalized ratio; APTT: activated partial thromboplastin time; AST: aspartate aminotransferase; ALT: alanine aminotransferase; CK: creatine kinase; CK-MB: creatine kinase-myocardial band; LDH: lactate dehydrogenase; eGFR: estimated glomerular filtration rate; BUN: blood urea nitrogen; IgG4: immunoglobulin G4

Test	Value	Reference range
WBC	5.51 x10^3/uL	4.00-11.00
MCV	93.5 fL	80-99.0
HGB	10.0 g/dL	13.0-17.0
PLT	205 x10^3/uL	130-400
PT	14.3 second	11.0-15.0
INR	1.05	0.78-1.12
APTT	34.7 second	32.0-45.1
D-Dimer	0.30 ug/mL	<0.5
Glucose	114 mg/dL	80-140
BUN	62 mg/dL	6.0-20.0
Creatinine	5.3 mg/dL	0.7-1.2
eGFR	12 mL/min/1.73m^2^	-
AST (GOT)	68 U/L	<40
ALT (GPT)	71 U/L	<41
CK	72 U/L	20-200
CK-MB	11.0 U/L	<25.0
LDH	264 IU/L	<250
Bilirubin T	0.2 mg/dL	0.0-1.2
Lipase	367 U/L	13-60
Sodium	136 mEq/L	136-145
Potassium	5.1 mEq/L	3.5-5.1
Troponin-T	0.037 ng/mL	0.000-0.014
Amylase	238 IU/L	28-100
Calcium	7.3 mg/dL	8.6-10.2
IgG4	15.40 mg/dL	3.0-201.00
HbA1c	5.80%	<5.7%
Triglycerides	132 mg/dL	<150

**Figure 1:**
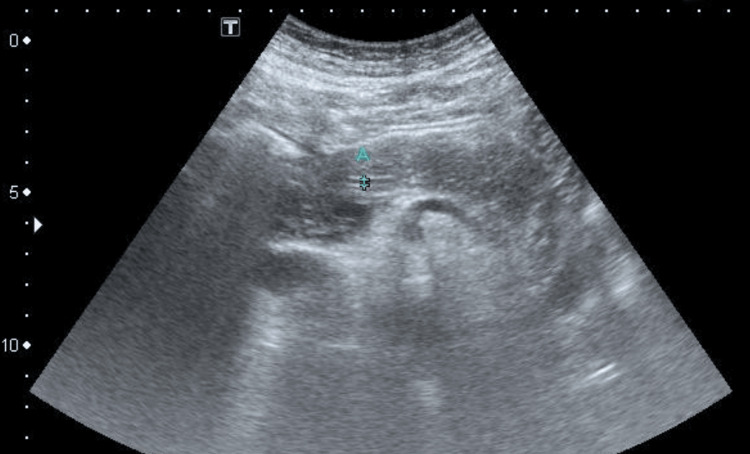
Abdominal ultrasonography demonstrating mildly heterogeneous pancreatic parenchyma and a prominent main pancreatic duct. Calipers at point A indicate measurement of the ductal diameter, approximately 2.3 mm.

On hospital day 1, the patient underwent an abdominal ultrasound examination, which revealed only slightly heterogeneous pancreatic parenchyma and a prominent main pancreatic duct measuring up to 2.3 mm with no enlargement, blurred border, fat stranding, or peripancreatic fluid collection (Figure 1). The patient was managed with supportive therapy, including fasting, fluid resuscitation, analgesia, and administration of gabexate mesilate. After two days of treatment, the patient’s abdominal pain markedly improved, and he was able to tolerate a low-fat soft diet. He was subsequently discharged in stable condition.

At follow-up, semaglutide was discontinued due to the episode of acute pancreatitis, and linagliptin monotherapy was initiated for glycemic control, considering the patient’s advanced chronic kidney disease and the limited availability of renally safe antidiabetic agents under such conditions. During the subsequent three months of outpatient follow-up, the patient remained free of recurrent abdominal pain.

## Discussion

The diagnosis of acute pancreatitis in this patient was established based on the Revised Atlanta Classification. In this case, the patient presented with acute-onset epigastric pain and a serum lipase level of 367 U/L (>6 times the upper limit of normal), fulfilling two of the three diagnostic criteria despite the absence of definitive radiologic findings. We acknowledge that abdominal ultrasonography may have limited sensitivity for early or mild acute pancreatitis, particularly in the absence of contrast-enhanced CT imaging, which was avoided in this case due to advanced renal dysfunction. This limitation is recognized to improve diagnostic transparency. Although the abdominal pain was intermittent and of moderate intensity, atypical presentations of acute pancreatitis, particularly mild or early-stage disease, have been well documented, and the absence of abdominal tenderness does not exclude the diagnosis. The patient’s rapid clinical improvement with supportive management further supports a diagnosis of mild acute pancreatitis.

Several alternative etiologies were carefully considered. Alcohol-related pancreatitis was deemed unlikely, as the patient had remained abstinent for more than five years, and there was no history of recent alcohol intake preceding symptom onset. Chronic alcohol-related pancreatitis typically manifests after sustained heavy intake with recurrent or progressive pancreatic injury, which was not observed in this patient. Similarly, while smoking is recognized as a risk modifier for pancreatitis, it is generally considered a contributing rather than initiating factor and does not readily explain an isolated acute episode with a clear temporal association following prolonged stable semaglutide exposure. Hypertriglyceridemia was excluded based on normal serum triglyceride levels, and IgG4-related disease was considered unlikely given a normal IgG4 concentration. There was no prior history of gallstones, and abdominal ultrasonography revealed no biliary dilatation or gallbladder pathology.

Given the patient’s advanced chronic kidney disease, the possibility of non-pancreatic hyperlipasemia was carefully considered. Although reduced renal clearance is known to cause mild elevations in serum lipase, elevations exceeding three times the upper limit of normal, particularly when accompanied by compatible abdominal symptoms, remain more suggestive of true pancreatic inflammation rather than isolated renal-related enzyme elevation. Importantly, this patient had a documented baseline lipase measurement during a prior hospitalization in December 2022 for chemotherapy-induced vomiting related to colon cancer treatment. At that time, his renal function was already significantly impaired (serum creatinine 3.6 mg/dL, estimated glomerular filtration rate 19 mL/min/1.73 m^2^), consistent with stage 4 chronic kidney disease; however, his serum lipase level was only mildly elevated at 70 U/L. This historical comparison suggests that advanced chronic kidney disease alone did not result in marked hyperlipasemia in this patient. In contrast, during the index presentation, the serum lipase level rose to 367 U/L, exceeding six times the upper limit of normal and coinciding with acute epigastric pain, supporting a diagnosis of true acute pancreatitis rather than renal-related enzyme accumulation.

Importantly, the patient had no prior history of pancreatitis during more than 25 years of diabetes management, including prior exposure to other glucose-lowering therapies. No recent dose escalation, interruption, or re-initiation of semaglutide occurred; however, delayed-onset drug-induced pancreatitis has been reported with GLP-1 receptor agonists, particularly in patients with complex comorbidities. The absence of recurrent symptoms following permanent discontinuation of semaglutide further supports a possible causal association. Other concomitant medications, including sulfonylureas, statins, beta-blockers, and antihypertensive agents, were reviewed. These agents have only rarely been implicated in acute pancreatitis, primarily through isolated case reports, and the patient had been receiving these medications stably for several years without prior pancreatic adverse events, making them less likely contributors in this case. Taken together, after systematic exclusion of alternative etiologies, semaglutide remains the most plausible precipitating factor for acute pancreatitis in this multimorbid patient.

As the first long-acting, once-weekly, subcutaneously administered GLP-1 RA with proven weight-reducing effects, semaglutide has been increasingly utilized in clinical practice, being prescribed not only for glycemic control but also for weight management and cardio-renal protection. Like other medicines, semaglutide may have drug-related side effects. The most common adverse effects are gastrointestinal symptoms, including nausea, vomiting, diarrhea, constipation, and dyspepsia, with an incidence of up to 74% [10]. These events are generally mild to moderate in severity, typically most pronounced during the dose-escalation phase, and tend to subside over time. Such adverse effects frequently lead to treatment discontinuation or dose reduction in some patients, with discontinuation rates reported at approximately 6-7%. The risk of hypoglycemia is low when semaglutide is used as monotherapy, but the risk increases when combined with insulin or sulfonylureas. Other uncommon adverse effects include injection-site reactions, hypersensitivity reactions, headache, dizziness, and reduced appetite. Currently, there is no definitive evidence indicating that semaglutide increases the risk of thyroid or pancreatic cancer [11].

Less common but clinically important complications include gallbladder diseases, such as cholelithiasis and cholecystitis, with an incidence of approximately 2-4%. The risk appears to increase in association with rapid weight loss, which may promote biliary cholesterol supersaturation and impaired gallbladder emptying. In addition, GLP-1 receptor agonists have been shown to reduce gallbladder motility, potentially leading to bile stasis and gallstone formation. Gallstone migration or biliary sludge may subsequently precipitate pancreatic duct obstruction and trigger acute pancreatitis. Beyond gallstone-related mechanisms, experimental and clinical studies have suggested that GLP-1 receptor activation may exert direct effects on pancreatic exocrine tissue, including altered acinar cell signaling and local inflammatory responses, although definitive causal pathways in humans remain incompletely understood [9]. These mechanisms may be particularly relevant in patients with multimorbidity, in whom impaired metabolic reserve, chronic kidney disease, or prolonged drug exposure could increase susceptibility to pancreatic injury.

According to current evidence, there is a relationship between semaglutide and acute pancreatitis, but semaglutide does not increase the overall risk of acute pancreatitis compared with placebo groups in large randomized controlled trials and real-world cohorts, and the incidence of acute pancreatitis remains low [12,13]. However, an important consideration in interpreting the available evidence regarding semaglutide and acute pancreatitis is the potential for patient selection bias in trials. Large randomized controlled trials, such as the Semaglutide Unabated Sustainability in Treatment of Type 2 Diabetes (SUSTAIN) programs and the Semaglutide Treatment Effect in People with obesity (STEP) programs, applied stringent inclusion and exclusion criteria. Patients with a history of malignant neoplasm in the previous five years, advanced chronic kidney disease, severe congestive heart failure, or significant hepatic dysfunction were typically excluded. Consequently, these “low-risk” trial populations are less likely to reveal the true adverse event rate in “high-risk” populations, including patients with multiple comorbidities. Supporting this notion, a recent retrospective case-control study of adults initiated on GLP-1 receptor agonists for weight management identified coronary artery disease/peripheral vascular disease and tobacco use as independent predictors of acute pancreatitis in this real-world setting, suggesting that underlying cardiometabolic disease and lifestyle factors may modify individual susceptibility to pancreatitis with GLP-1RA therapy [14]. Such observational evidence underscores the potential for heterogeneous risk profiles that may not be fully captured in randomized trials focused on healthier subjects.

What’s more, in real-world prescribing patterns, clinicians tend to take the patient’s adherence, motivation, and overall health assessment into consideration rather than simply following expert guidelines [15]. Therefore, according to a recent large-scale US insurance database and multi-center study, the actual users of semaglutide are mostly high-income, White, female, living in urban areas, and most have commercial insurance, indicating that financial ability and insurance coverage are important factors affecting prescriptions [16].

This dual bias suggests that the true risk of pancreatitis in high-risk populations may be underestimated. In our case, patients with a recent history of cancer treatment and multiple underlying comorbidities warrant heightened vigilance with respect to safety considerations. Patients with multimorbidity are defined as individuals who have two or more chronic conditions simultaneously. Such patients are highly prevalent in clinical practice, often emerging earlier among older adults and socioeconomically disadvantaged populations [17]. Multimorbidity is closely associated with higher mortality, functional impairment, reduced quality of life, and increased health care utilization [18]. Notably, these groups have often been excluded from semaglutide clinical trials and, due to economic barriers, have poorer access to treatment in real-world settings. Consequently, retrospective studies may face challenges of biased effect estimation and limited external validity when evaluating outcomes in these populations. To better quantify this potentially under-recognized risk, future research should leverage real-world data sources, such as large administrative health databases, disease-specific registries, or multicenter prospective observational cohorts, with deliberate inclusion of patients with multimorbidity, advanced chronic kidney disease, or recent malignancy. Study designs incorporating active comparator groups, longitudinal follow-up, and stratified analyses by comorbidity burden may help clarify heterogeneous risk profiles that are not adequately captured in randomized clinical trials.

Currently, there is limited research on the use of semaglutide in patients with multimorbidity. Dagher et al. [19] described a fatal event in a 74-year-old male with a medical history of type 2 diabetes, atrial fibrillation, coronary artery disease, and obesity. The patient was admitted to the intensive care unit from the emergency department and, within two days, developed respiratory failure and severe shock, ultimately receiving palliative care and passing away. Turunen et al. [20] reported a 61-year-old man with a complex medical history, including gastroesophageal reflux disease, irritable bowel syndrome, sleep apnea, type 2 diabetes mellitus, dyslipidemia, generalized anxiety disorder, and bipolar type 2 disorder, who suffered from semaglutide-associated gastric pneumatosis. Future studies should address several key research gaps, particularly in patients with multimorbidity. These include more precise characterization of safety outcomes in high-risk populations, evaluation of optimal dosing strategies and duration of exposure in patients with impaired organ reserve, and identification of appropriate clinical or laboratory monitoring parameters for early detection of adverse events. Addressing these gaps may help guide individualized risk stratification and safer use of semaglutide in real-world clinical practice.

## Conclusions

This case highlights that although semaglutide has demonstrated a favorable safety profile in clinical trials and real-world studies, its safety in patients with multimorbidity remains incompletely understood. As semaglutide use continues to expand, clinicians should remain vigilant for potential drug-related adverse events in higher-risk patients. In everyday practice, such vigilance may include careful assessment of baseline risk factors (e.g., prior pancreatitis, advanced chronic kidney disease, cardiovascular disease, or smoking history), patient education regarding early gastrointestinal warning symptoms, and prompt evaluation of abdominal pain with appropriate laboratory testing. Periodic reassessment of the ongoing risk-benefit balance may be particularly important in patients receiving long-term therapy. Future studies focusing on these populations are warranted to better inform individualized monitoring and safer use of semaglutide.
